# Microscopic evaluation of induced tooth movement after subluxation trauma: an experimental study in rats

**DOI:** 10.1590/2176-9451.19.1.092-099.oar

**Published:** 2014

**Authors:** Mauro Carlos Agner Busato, Alex Luiz Pozzobon Pereira, Celso Koogi Sonoda, Osmar Aparecido Cuoghi, Marcos Rogério de Mendonça

**Affiliations:** 1 Assistant professor, State University of Western Paraná (UNIOESTE).; 2 Adjunct professor, Department of Dentistry, Federal University of Maranhão (UFMA).; 3 Full professor, Department of Orthodontics, State University of São Paulo (UNESP).; 4 Full professor, Department of Orthodontics, State University of São Paulo (UNESP).

**Keywords:** Orthodontics, Tooth injuries, Periodontium

## Abstract

**Objective:**

The objective of this study was to assess the histological alterations that occurred in the periodontal area of rat molars submitted to induced tooth movement (ITM) right after an intentional trauma (subluxation).

**Methods:**

Forty adult male Wistar rats (*Rattus norvegicus albinus*) were selected. The animals were divided into eight groups (n = 5), according to the combination of variables: Group 1 - control (neither trauma nor ITM); Group 2 - ITM; Groups 3, 4, 5 and 6 - dentoalveolar trauma groups corresponding, respectively, to 1, 3, 8 and 10 days after trauma; Groups 7 and 8 - the animals' molars were subjected to a 900 cN impact and, one and three days after the trauma event, tooth movement was induced. The rats' maxillary first molars were mesially moved during seven days with a closed coil (50 cN). After the experimental period of each group, the animals were sacrificed by anesthetic overdose and the right maxillas were removed and processed for histological analysis under light microscopy.

**Results:**

In the animals of group 3, 4, 5 and 6, the histological alterations were not very significant. Consequently, the effect of induced tooth movement right after a subluxation event (groups 7 and 8) was very similar to those described for Group 2.

**Conclusion:**

There was no difference in the quality of periodontal repair when ITM was applied to teeth that had suffered a subluxation trauma.

## Abstract (pt)

**Objetivo:**

avaliar as alterações histológicas ocorridas na área periodontal de molares de ratos submetidos à movimentação dentária induzida (MDI), logo após um trauma intencional (subluxação).

**Métodos:**

quarenta ratos Wistar machos adultos (*Rattus norvegicus albinus*) foram selecionados. Os animais foram divididos em oito grupos (n = 5), de acordo com a combinação das variáveis: Grupo 1 - controle (sem trauma e sem MDI); Grupo 2 - MDI; Grupos 3, 4, 5 e 6 - grupos de trauma dentoalveolar correspondendo, respectivamente, para 1, 3, 8 e 10 dias após o trauma; Grupos 7 e 8 - os molares murinos foram submetidos a um impacto de 900cN e, de um e três dias após o evento trauma, o movimento do dente foi induzido. Os primeiros molares superiores dos animais foram movidos mesialmente durante sete dias, com uma mola fechada (50cN). Após período experimental de cada grupo, os animais foram sacrificados por overdose anestésica e as maxilas direitas foram removidas e processadas para análise histológica qualitativa.

**Resultados:**

nos animais dos grupos 3, 4, 5 e 6, as alterações histológicas não foram muito significativas. Consequentemente, o efeito do movimento dentário induzido logo após um evento de subluxação (grupos 7 e 8) foi muito semelhante ao descrito para o grupo 2.

**Conclusão:**

não houve diferença na qualidade do reparo periodontal quando a MDI foi aplicada aos dentes que sofreram um trauma de subluxação.

## INTRODUCTION

According to the classification of the World Health Organization (WHO),^1^ dentoalveolar trauma may affect the teeth, supporting tissues, gingiva and the oral mucosa. From an orthodontic point of view, the main concern is about trauma caused to the periodontal tissues, including the alveolar bone, periodontal ligament and root cementum. This is because the success of orthodontic therapy depends on the normality of these tissues, since tooth movement is directly associated with the supporting peiodontium.^2-5^

Subluxation is the most common trauma that affects the supporting periodontium, representing one fourth of all traumas that involve injury to the periodontal ligament.^6^ In subluxation, the tooth is not displaced from its initial position, and after trauma it will have a certain degree of mobility.^1^

After subluxation trauma, the histological events involve, in the first few hours, hemorrhage in the periodontal ligament space, lesion in periodontal fibers and cell death. Afterwards, hyalines and inflammatory mediators, which induce bone and root resorption, will appear. Ten days after trauma, with less concentration of inflammatory products, periodontal repair begins.^1,7^

The protocols of the International Association of Dental Traumatology,^8,9,10^ disclosed in 2007, present a consensual view of several researchers and clinicians from different specialties about the care and procedures performed in traumatized teeth. This guide suggests treatment procedures for different types of traumatism, indicating that in the event of subluxation, there is no need for specific treatment and the involved teeth must be followed-up for at least three months before being submitted to orthodontic forces.^8,9,10^ This is the current paradigm with regard to subluxated teeth: orthodontic force is considered an additional trauma to the periodontium, thus, the professional must be cautious when recommending it.^11^

In opposition to the state of the art with regard to movement of traumatized teeth, other authors suggest that movement of luxated teeth may relieve the areas of compression in the periodontium and pulp, and, therefore, facilitate the repair of these tissues.^3,12^ As a secondary consideration, Mine et al^3^ inferred that calculated mechanical stimuli, such as orthodontic forces, would be important so that after injury, the process of migration and proliferation of periodontal ligament cells would occur, preventing the occurrence of ankylosis and inflammatory root resorption.

Nevertheless, the real histological effect of applying an orthodontic load on a recently traumatized tooth has not been experimentally established.^1,13-16^ Furthermore, the relationship between time and the movement of a traumatized tooth also needs to be experimentally established for the periodontal tissues. In other words, there is a lack of scientific evidence to support the clinical procedures.^5^

Thus, this study assessed the histological alterations that occurred in the periodontal tissue of rat molars submitted to induced tooth movement (ITM) right after an intentional trauma (subluxation).

## MATERIAL AND METHODS

The research project was independently reviewed and approved by the Animal Research Ethics Committee of the School of Dentistry of Araçatuba, State University of São Paulo (UNESP, Brazil). All guidelines regarding the care of animals used during research were strictly followed.

Forty male Wistar rats (*Rattus norvegicus albinus*) weighing between 250 and 350 g were selected for the study. The animals were housed in plastic cages under controlled climate conditions (12h light / 12h dark; thermostatically regulated room temperature), were fed with standard solid food (Ração Ativada Produtor; Anderson & Clayton S.A. Indústria e Comércio, São Paulo/SP, Brazil) and water *ad libitum*.

All experimental procedures were performed under anesthesia. The animals received an intramuscular injection of xylazine hydrochloride (Dopaser^®^; Caleir S.A., Barcelona, Spain; 0.03 ml/100 g body weight) and ketamine hydrochloride (Vetaset^®^; Fort Dodge Animal Health, Overland Park, KS, USA; 0.07 ml/100 g body weight).

The animals were divided into 8 groups (n = 5). In Group 1 (control group - normal periodontium), animals were monitored throughout the whole experiment without induction of any treatment. After the experimental period, they were submitted to euthanasia. In Group 2 (induced tooth movement group), a device used to induce tooth movement was placed in the animals, and after seven days of movement the animals were submitted to euthanasia.

In Groups 3, 4, 5 and 6 (trauma groups corresponding, respectively, to 1, 3, 8 and 10 days after trauma), the animals received an intentional dentoalveolar trauma on the occlusal surface of the maxillary first molar and after time intervals of 1, 3, 8 and 10 days, respectively, they were submitted to euthanasia. In Group 7 (one-day movement trauma group), a device used to induce tooth movement was placed one day after dentoalveolar trauma, and after seven days of movement, the animals were submitted to euthanasia. In Group 8 (three-day movement trauma group), a device used to induce tooth movement was placed three days after dentoalveolar trauma, and after seven days of movement, the animals were submitted to euthanasia.

**Figure 1:**
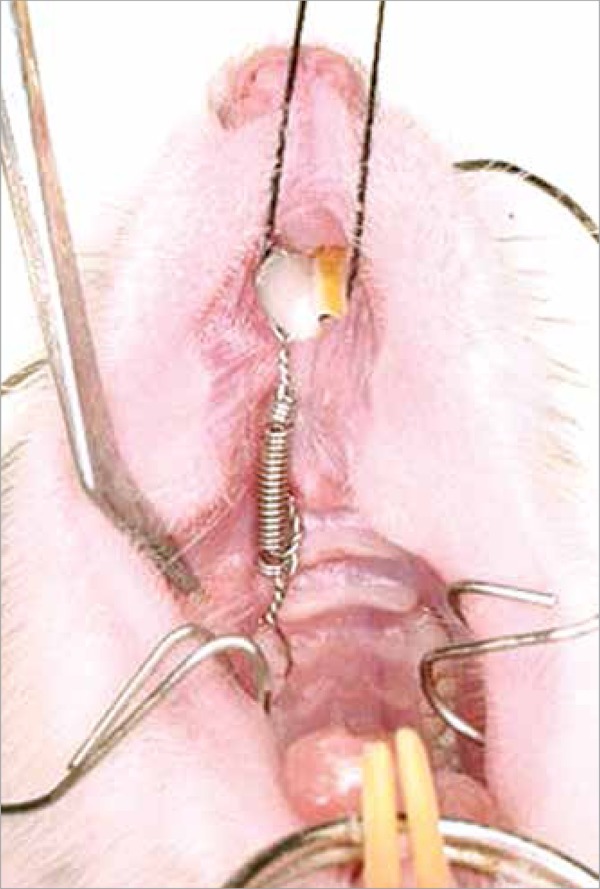
Tooth movement device proposed by Heller and Nanda.^17^ Extended coil (50 cN).

In the groups of animals submitted to induced tooth movement (Groups 2, 7 and 8), a device similar to the model proposed by Heller and Nanda^17^ (Fig 1) composed of a closed section coil (Morelli, Sorocaba, São Paulo, Brazil - code 35.20.061) and two 0.25 mm wire ligatures (Morelli, Sorocaba, São Paulo, Brazil - code 55.01.210) were placed on the right maxillary first molar in order to promote molar mesial movement by applying controlled force of 50 cN, measured with the aid of a tensiometer (Zeusan Exporting Ltda Campinas, São Paulo, Brazil).

**Figure 2:**
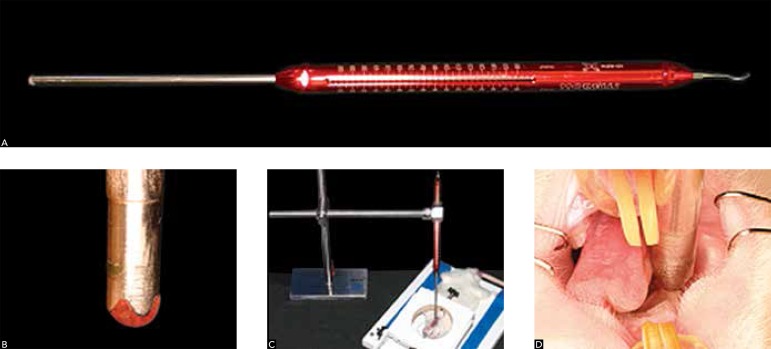
Device used to promote experimental dentoalveolar trauma: A) Tensiometer used to assess the load. B) Tensiometer active tip adapted with acrylic resin. C) Tensiometer properly adjusted to the rat's molar. D) Tensiometer extremity adjusted to the vestibular crest of the right maxillary first molar.

Animals in the groups submitted to dentoalveolar trauma (Groups 3, 4, 5, 6, 7 and 8) received a standardized impact of 900 cN directed to the occlusal surface of the right maxillary first molar using an adapted tensiometer (Morelli, Sorocaba, Sao Paulo, Brazil - code 75.02.006) in order to maintain the same intensity and direction of force application, as described by Pereira et al^18^ (Fig 2).

Immediately after the experimental period of each group, the animals were sacrificed by anesthetic overdose through peritoneal injection. The right maxillas were removed, fixed in 10% buffered formalin for 24 hours and decalcified in 10% EDTA solution for 5 weeks. After decalcification, the specimens were embedded in paraffin and serial histological sections 6 µm thick were cut from the mesial root of the maxillary right first molar (including the surrounding tissues) in a longitudinal (mesiodistal) direction following the long axis of the tooth. For histological analysis, sections from the middle portion of the root were collected, stained with hematoxylin and eosin and observed under light microscopy (Carl Zeiss do Brasil Ltda., São Paulo/SP - Brazil). The areas in the cervical, middle and apical thirds of the mesial root of the first maxillary molar were selected for histological description under 73 and 170x magnification. The following tissues were considered for analysis: alveolar bone, cementum and periodontal ligament.

## RESULTS

In Group 1 (with no trauma or movement), the periodontal ligament was rich in collagen fibers and fibroblasts. The collagen fiber bundles were disposed horizontally in the cervical region, while in the middle and apical thirds they were obliquely placed. In the periapical and furcation region, these fibers were irregularly disposed. Close to the apex, the dentin was covered by primary cementum which was covered by a thin layer of secondary cementum. The alveolar walls were rich in osteoblasts and osteocytes.

**Figure 3:**
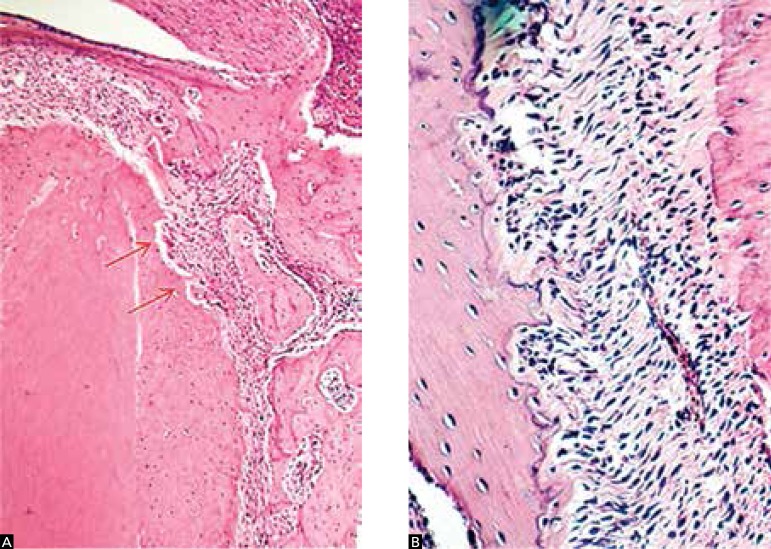
Groups 5 and 6. A) Apical third of the distal surface of the root with surface cementum resorption (arrows) - mag. 63x. B) Alveolar crest. Active bone resorption, with the presence of clasts - mag. 160x.

**Figure 4:**
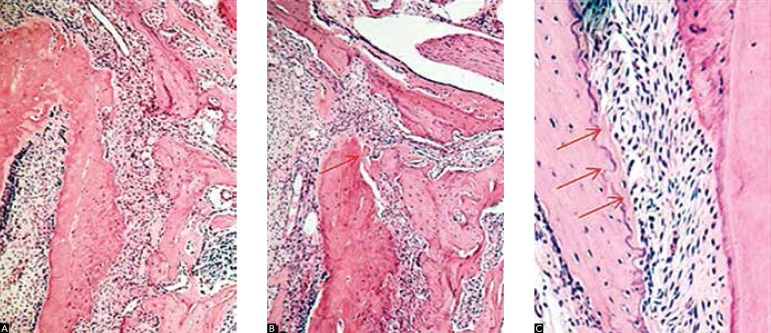
Group 2. A) Apical third of the distal surface of the root with periodontal ligament (fibroblasts and collagen fibers) irregularly disposed - mag. 63x. B) Points of contact between root and alveolar surfaces in the apical third of the distal surface of the root (arrow) - mag. 63x. C) Alveolar crest with neoformed bone tissue (arrows) - mag. 160x.

Animals submitted to dentoalveolar trauma only (Groups 3, 4, 5, 6) presented microscopic characteristics that were similar to Group 1, except for some animals showing points of superficial root resorption and some points of resorption in the alveolar walls, with osteoclasts near the resorbed area (Fig 3). In the animals submitted to induced tooth movement (Group 2), the root surface was intact throughout the entire extension of its mesial portion. A layer of cementoblasts-like cells was observed in the cementum that covers the root surface. Near this structure, the extremities of the collagen fibers of the periodontal ligament were found. In one specimen, small points of proximity between the cementum and the alveolar bone were found in the distal surface of the root only in the apical third. In all the specimens, the periodontal ligament was rich in collagen fibers and fibroblasts. In the cervical third of the mesial surface and in the apical third of the root, the fibers had no defined organization. In this region, an infiltrate of lymphocytes and histiocytes in the fibrous conjunctive tissue was also found in some specimens. In three specimens, some points of proximity between the cementum and the alveolar bone were found in the apical third of the distal surface of the root. In the other areas, the fibers were obliquely disposed and inserted in the cementum surface on one side and in the bone surface on the other. In the alveolar bone crest, some points of bone resorption were found in one specimen. In other specimens, these points were characterized by small bone cavities with no clasts and with neoformed bone tissue (Fig 4).

**Figure 5:**
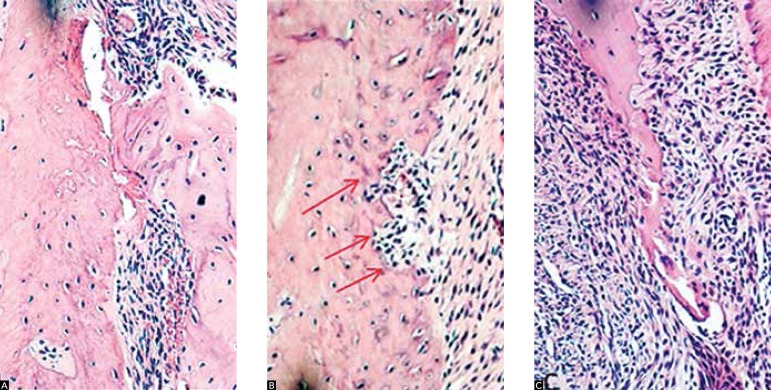
Groups 7 and 8. A) Apical third of the root, hyaline areas - mag. 160x. B) Points of root resorption (middle third of the root - arrows) - mag. 160x. C) Fracture of the alveolar crest and osteoclasts - mag. 160x.

In Groups 7 and 8, corresponding to the teeth that were traumatized and moved one and three days after dentoalveolar trauma, the root surface on the mesial side was intact throughout its entire extension. A layer of cementoblasts-like cells was found in the cementum layer that covers the root surface. In five specimens from Group 7 and in two specimens from Group 8, areas of surface resorption were found, especially in the apical portion on the distal side of the mesial root. In two specimens (Group 7), deeper areas of cementum-dentin resorption were found in the middle third on this side of the root. In one of them, an area of ankylosis was also detected in the middle third, and in two specimens, an area of hyaline degeneration was found, one in the mesial cervical region and another in the distal apical portion. Group 8 showed an area of greater proximity of the root surface to the alveolar bone wall in the apical third in three specimens. In this space, the presence of a small area of hyaline degeneration was found. In all specimens from Group 7 and 8, the periodontal ligament was rich in collagen fibers and fibroblasts. The fibers were obliquely disposed in comparison to the root surface, and were inserted into the cementum surface on one side and into the alveolar bone wall on the other. In four specimens from Group 7 and three from Group 8, several points of bone resorption were observed in the cervical third of the alveolar wall on the mesial side of the root. In the other areas, bone tissue was intact in its whole extension (Fig 5).

## DISCUSSION

The characteristics of tissues under induced tooth movement in normal rats have been widely studied and the morphological features described were similar to those seen in human teeth.^19,20,21^ Conversely, descriptions of the aspects of induced tooth movement in teeth under the influence of periodontal trauma are rare.^22,23,24^

Turley et al^22,23^ and Gomes et al^24^ induced movement of traumatized teeth. They worked with an experimental model comprising dog premolars and incisors with more severe levels of trauma (intrusive luxation). The option of studying subluxation was based on the fact that this type of trauma is very prevalent among injuries of the periodontal ligament.^6^ Furthermore, despite the fact that the histological events after luxation have been widely described in the literature, there is a lack of studies with regard to how repair occurs in cases of subluxated teeth.

Therefore, with respect to the periodontal ligament of the assessed groups, a similarity between the architectural organization of the periodontal fibers in the dentoalveolar trauma groups (Groups 3, 4, 5 and 6) and in the normal periodontium group (Group 1) was observed, thus showing that the magnitude of the trauma applied can be considered light. With regard to the groups that received orthodontic movement, either in the presence (Groups 7 and 8) or absence of trauma (Group 2), a similarity in the microscopic events was also observed with absence of a definite organization of collagen fibers, and presence of signs of inflammatory events, especially in the mesial cervical region and distal apical portion of the root. This probably occurred because, in these areas, the periodontal ligament is compressed during molar inclination as a result of tooth movement.

Although there have been divergences with regard to experimental time, type of trauma and the fact that the tooth movement inducing device was reactivated, Turley et al^23^ observed that in the teeth that suffered slight intrusions (0.5 to 1 mm) and were orthodontically extruded afterwards, inflammatory root resorption occurred and this result was similar to Groups 7 and 8 of this study, in which points of surface resorption were observed in seven out of ten animals, particularly in the apical portion of the distal side of the mesial root that was an area of periodontal ligament compression. These results of superficial and inflammatory root resorption were also detected in the groups of animals submitted to dentoalveolar trauma (Groups 3, 4, 5 and 6), although in limited areas and of less magnitude. Deeper areas of cementum-dentin resorption were also found in the middle thirds of the root of two specimens from Group 7, whereas and in one of them, an area of proximity between the cementum and the alveolar bone was found. Gomes et al^24^ also found similar results in dogs. They observed extensive lacunar resorption that reached the dentin, mainly in the cervical third, but with great repair activity and no evidence of active root resorption. In the induced tooth movement group (Group 2), areas of proximity were also found between the cementum and the alveolar bone in the apical third of the distal surface of the root. It seems that the contact between the cementum and the alveolar bone in Groups 7 and 8 is more related to induced tooth movement than to the effect of the trauma, since those events were also observed in Group 2. This aspect was not found in the groups submitted to trauma (Groups 3, 4, 5 and 6).

As found by Turley et al^22^ and Gomes et al^24^, areas of bone resorption with the presence of multinucleated cells in the Howship lacunae were also observed in seven specimens from Groups 7 and 8. In the group submitted to induced tooth movement (Group 2), the same resorption lacunae were also found. Once again, when trauma was associated with movement (Groups 7 and 8), the events of alveolar bone wall repair were similar to those of Group 2.

Considering the histological events of the supporting periodontium after subluxation trauma and induced tooth movement, there seems to be no objective reasons to wait after this type of trauma to start orthodontic treatment. The hyalinization of the periodontal ligament as well as the root and bone resorptions observed were similar in the induced tooth movement groups (Groups 2, 7 and 8) in such a way that the application of a controlled orthodontic force on a tooth that suffered subluxation trauma is not contraindicated. In other words, under the experimental conditions of the present study, the follow-up time of a traumatized tooth recommended by the literature,^6,8,9,10^ before orthodontic movement is induced, does not seem to be true in the case of teeth that suffered subluxation.

## CONCLUSION

Based on the methods and the results of this study, it is reasonable to conclude that there was no difference in the quality of the periodontal tissue response when induced tooth movement was applied in one and three days after an event of subluxation.
